# Commentary: Systematic assessment of duration and intensity of anodal transcranial direct current stimulation on primary motor cortex excitability

**DOI:** 10.3389/fnhum.2016.00439

**Published:** 2016-08-30

**Authors:** Claire J. Hanley

**Affiliations:** Department of Psychology, Swansea UniversitySwansea, UK

**Keywords:** transcranial electrical stimulation (tES), transcranial direct current stimulation (tDCS), neuromodulation, cortical excitability, inter-individual variability, null results

As a pertinent addition to the literature, the study highlights several concerns currently facing those in the transcranial electrical stimulation (tES) community.

## Standing out from the crowd

Tremblay et al. (2016) investigated modulations of corticospinal excitability within motor cortex as induced by transcranial direct current stimulation (tDCS). Having delivered 1 and 2 mA stimulation for durations of 10 or 20 min, they assessed the outcomes by means of motor evoked potentials (MEPs). This may be regarded as “business as usual” by many researchers. Indeed, there is nothing ground-breaking about the paradigm but therein lies its appeal. The article represents a systematic account of tDCS effects resulting from some of the most commonly used protocols, which in an increasingly discordant field was well overdue.

tDCS has become increasingly popular as a result of research by Nitsche and Paulus (2001), which documented the existence of sustained after-effects in humans. However, the explosion of studies since this time has had its drawbacks—primarily, the heterogeneity in stimulation parameters (e.g., current intensity, duration, electrode size) that have been adopted to address a multitude of research objectives. The lack of consistency means that researchers, conducting successful and unsuccessful studies alike, are unable to infer which specific components of their protocols are particularly influential with regard to the observed results. This is extremely problematic, especially where stimulation is applied to aid in rehabilitation or attenuate the symptoms of neurological and psychiatric conditions (Brunoni et al., 2012).

In this context, the article by Tremblay et al. represents a milestone for tES research. Systematic studies of tDCS effects are currently in the minority and researchers are largely unaware of the influence subtle variations in stimulation parameters are likely to have on results. Those studies that do exist have utilized relatively complex designs and atypical stimulation parameters (weak current strengths, novel stimulation intervals and/or small electrodes e.g., Batsikadze et al., 2013; Monte-Silva et al., 2013; Bastani and Jaberzadeh, 2014; Chew et al., 2015), which limits their applicability to the field as a whole. It is crucial that the efficacy of more widely used protocols is fully assessed to determine whether they are capable of generating replicable results. The paper by Tremblay et al. is particularly relevant for this reason as it offers a timely evaluation of standard practice.

## Opening the “file drawer”

It cannot be ignored that the article reflects a series of non-significant results. None of the four assessed intensity/duration pairings altered corticospinal excitability in a manner that was statistically meaningful. Prominent inter-individual differences in response rates were likely to have contributed to these outcomes, with non-responders constituting up to 80% of the sample, but what does this variability mean for the future of tES?

One may assess these findings in the context of recent meta-analyses, which demonstrate a lack of consistent effects on behavioral and neurobiological metrics, and decide to view them as a further blow to a field already marred by controversy (Horvath et al., 2015a,b). To do so would be to discredit a technique with vast potential and disregard the emerging evidence regarding sustained benefits in clinical populations (Khedr et al., 2014; Allman et al., 2016). That is not to say that these non-significant results should be overlooked. Traditionally, null results are reviled, particularly where they represent a deviation from the norm. In such circumstances atypical results are often attributed to methodological flaws, however, the rigorous design of the study by Tremblay et al. makes the outcomes hard to ignore. The study is clearly based on a-priori knowledge, possesses ample sample sizes and features a critique of potential predictive factors e.g., baseline excitability and inter-trial differences in MEP amplitudes. While the article asserts that response variability is very much an issue in tES research (supporting the findings of Wiethoff et al., 2014 and López-Alonso et al., 2014), crucially, what it infers is not to abandon the neuromodulation technique but the need for a dialogue regarding best-practice.

Advocating the publication of non-significant results, like those of the article in question, will inevitably assist in the reformation of tES research—especially where they originate from methods-driven investigations (Hanley et al., 2015). Null results, far from representing failure, should encourage researchers to learn from experience and refine methodology. Such optimization is imperative for tES as much of the inconsistency that plagues the current research climate can be attributed to poorly designed experiments; characterized by illogical hypotheses, generated in the absence of biologically relevant information (Bestmann et al., 2015). By focusing on improving basic research principles and ensuring good experimental design, variability can largely be controlled and accounted for. Of course, parameters that elicit the desired effect in some individuals may not be effective in others. This is likely to vary between states of health and disease, and could mean a uniform approach is not always appropriate (Kuo et al., 2014). As a beacon of best-practice, the study by Tremblay et al. highlights the fundamental need for optimization of stimulation protocols. Accordingly, part of good research practice will be to systematically determine the key to producing reliable and reproducible modulations of excitability, which may involve titrating stimulation for a particular population or even specific individuals.

## A step in the right direction

A number of issues concerning the validity of tES research are brought to light by Tremblay et al. (2016). As outlined by the authors themselves, current approaches lack sensitivity and place limitations on the reproducibility of effects. Their article signals the need for a shift in the priority of researchers, who are encouraged to acknowledge the power of methodological integrity and go back to basics before the field can evolve. This is essential if tES is to be taken seriously, such that it can be considered a tool of clinical relevance. For these reasons, the focus of this commentary should be regarded as the foundation on which to build the future of tES.

## Author contributions

The author confirms being the sole contributor of this work and approved it for publication.

### Conflict of interest statement

The author declares that the research was conducted in the absence of any commercial or financial relationships that could be construed as a potential conflict of interest.
